# Needle visualization during ultrasound-guided puncture: image optimization

**DOI:** 10.1590/1677-5449.202300382

**Published:** 2023-07-17

**Authors:** Augusto Cézar Lacerda Brasileiro, Aeudson Víctor Cunha Guedes e Silva, Ariana Lacerda Garcia, Beatriz Ribeiro Coutinho de Mendonça Furtado, Frederico Augusto Polaro Araújo, Laís Nóbrega Diniz, Leonardo César Maia e Silva, Lorena Agra da Cunha Lima

**Affiliations:** 1 Faculdade de Medicina Nova Esperança - FAMENE, João Pessoa, PB, Brasil.

**Keywords:** needle, ultrasound, angle, needle visualization software, agulha, ultrassom, ângulo, software de visualização de agulha

## Abstract

**Background:**

Obtaining an adequate image of the needle by ultrasound reduces complications resulting from punctures, increasing patient safety and reducing hospitalization costs.

**Objectives:**

To verify human perception in relation to number of pixels, while also identifying the best puncture angle and which needle should be used, and to evaluate whether there is a difference if needle visualization software is used.

**Methods:**

20 images were analyzed by 103 students who classified them as being sufficient or insufficient and were compared with the quality observed using photoshop. We evaluated whether there were differences between puncture angles of less than 45º and more than 45º, between IV catheter and introducer needles, and between images obtained with and without visualization software.

**Results:**

There was a higher percentage of sufficient ratings for images those that had more than 60 pixels and when the puncture angle was less than 45º, with significant associations between students’ evaluations and each of these groups (p < 0.001). The percentages of images classified as sufficient were higher for images in which a IV catheter was used and also higher for those using the needle visualization software, with significant associations between the results for students’ classifications and each of these groups (p < 0.001).

**Conclusions:**

The human eye classifies an image as sufficient according to higher numbers of pixels. Images of punctures at angles smaller than 45º in relation to the surface, of punctures performed with a IV catheter, and when using specific visualization software are also better detected by the human eye.

## Resumo

**Contexto:**

A obtenção de uma imagem adequada da agulha pelo ultrassom diminui complicações decorrentes de punções, trazendo segurança para os pacientes e diminuindo custos com hospitalização.

**Objetivos:**

Verificar a percepção do olho humano em relação aos *pixels*, identificar qual o melhor ângulo da punção e qual agulha deve ser utilizada e avaliar se há diferença com o uso de *software* de visualização de agulha.

**Métodos:**

Vinte imagens foram analisadas por 103 alunos, que as classificaram como sendo suficientes ou insuficientes, e comparadas com a qualidade observada pelo photoshop. Avaliou-se se havia diferença entre punções com menos de 45º e mais de 45º, entre cateter EV e agulha introdutora e entre imagens obtidas com e sem *software* de visualização.

**Resultados:**

Houve um percentual mais elevado de imagens suficientes entre aquelas que tinham mais de 60 *pixels* e quando o ângulo era menor que 45º, com associação significativa entre a classificação das avaliações pelos alunos e cada um desses grupos (p < 0,001). O percentual de imagens suficientes foi maior nas imagens realizadas com cateter EV e naquelas que utilizaram o *software* de visualização da agulha, ocorrendo associação significativa entre os resultados da classificação pelos alunos e cada um desses grupos (p < 0,001).

**Conclusões:**

O olho humano classifica a imagem como sendo suficiente de acordo com a maior quantidade de *pixels*. Imagens puncionadas com ângulos menores que 45º em relação à superfície, realizadas com cateter EV e utilizando *software* específico de visualização também são mais bem detectadas pelo olho humano.

## INTRODUCTION

Complications arising from punctures performed using anatomical references only can be avoided by gaining better understanding of the parameters for obtaining the best image of the needle using Ultrasonography (USG), thereby increasing safety for professionals and comfort for patients and reducing hospitalization costs.^1,2^

USG visualization can be improved or impaired depending on the parameters of the USG device itself and the individual characteristics of the needle.^3^ In addition, the angle of the needle in relation to the sound beam and the use or not of specific visualization software can also interfere with the quality of the images.^4-6^

At the time of puncture, the needle will form an angle in relation to the USG beam and in relation to the surface to be punctured. Since it is a highly reflective structure, when the waves produced by the probe hit the needle close to a right angle, a large proportion return to the probe, generating a good quality image. However, as the puncture angle becomes steeper in relation to the surface, the waves are reflected, but do not return to the probe, forming a worse quality image or even making the needle invisible.^7,8^

The resources available in the USG settings, the characteristics relevant to the composition of the needle (material, diameter, and length), and also the puncture angle in relation to the surface all affect reflection of the waves and change the quality of the image.^8,9^

Models used for training offer good resemblance to human tissues. Due to their ease of handling and low cost, models made from gelatin are mainly used.^10,11^

This study aims to verify the perceptions of medical students with little or no experience of USG guided puncture in relation to the number of pixels seen using specific software (photoshop), identifying in which situations images are better evaluated by the students, in relation to the angle between the needle and the surface to be punctured and which needle should be used for puncture, and also to evaluate whether there is a difference when needle visualization software is used.

## METHODS

This is an observational, cross-sectional, and analytical study. All students who were completing the fourth year of the FAMENE medical course were selected. We decided to consider all fourth-year medical students at FAMENE because we targeted a sample that had little or no contact with the ultrasound-guided access procedure. Students who did not want to participate in the research were excluded. The study was approved by the institutional Ethics Committee (58221622.6.0000.5179 - 5.860.556) and all participants were invited to participate in the research and signed a Free and Informed Consent Form.

We found a very small number of similar studies, which made it impossible to calculate the sample size in advance. However, we calculated the power of our sample. For the comparisons of the percentages of satisfactory results, with an error of 5% and the results contained in the tables of the article, the minimum sample power was 99.4%, showing that the sample size was sufficient. Power calculations were performed using G*Power software, version 3.1.9.2.

The students received an explanation and watched a video showing an ultrasound-guided vascular puncture, in which the needle was visualized in the longitudinal direction along its entire path into the lumen of the vessel. Afterwards, the research images were projected onto a screen and students were given enough time for everyone to classify them as being sufficient or insufficient to enable the same procedure observed in the video to be performed. The students therefore did not know which variables we were evaluating, only having contact with the images.

A 9L linear probe was used at a frequency of 10Mhz with a General Electric Logiq E R7 ultrasound machine with the settings adjusted (Gain, Time Gain Compensation, focus, depth, and dynamic range). A total of 10 images were acquired with Needle Recognition viewing software and 10 images were acquired without.

Two different 18 Gauge (G) needles were compared. One was a IV catheter (siliconized stainless steel needle), manufactured by Disposafe Health and Life Care - India (Anvisa nº 80808480018), and the other was an introducer needle (316L stainless steel) from the puncture kit for the Dignity Port chemotherapy portal system, manufactured by Medical Components - United States of America and Martech Medical Products - Mexico (Anvisa 10312210021).

**Figure 1:**
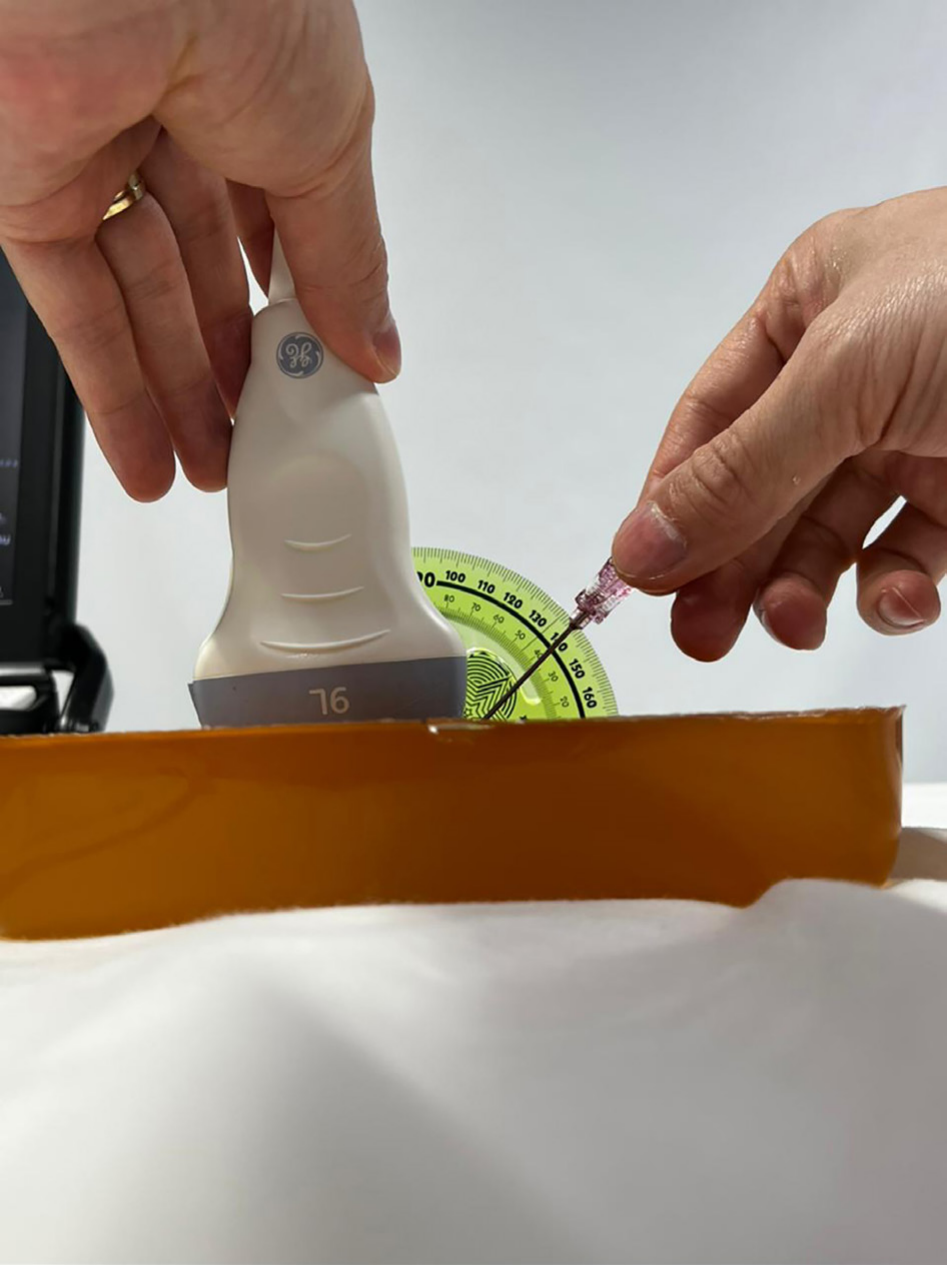
Puncture in gelatin model in the in-plan direction.

Punctures were performed at angles smaller than and greater than 45º in relation to the surface of the Phantoms (gelatin model) in the in-plan direction, using a protractor to measure the angles (Figure 1).^12^ The phantom models were homemade, containing water, gelatin, bidistilled glycerin, and sugar-free Metamucil. The mixture was boiled and allowed to cool in a container measuring 18 × 10 × 6 cm.^11,13^

All images were acquired by the same researcher. When the images were captured, they were also imported to Photoshop to confirm whether the angle was smaller or larger than 45º.

The images were analyzed using Adobe Photoshop (Version 21.2.2) by an evaluator who was not involved in the research. For the gray scale, we considered 0 (black) and 255 (white).^8,9^

### Data analysis

Data were analyzed descriptively using absolute and percentage frequencies. Pearson’s chi-square test was used to assess associations between two categorical variables, or Fisher’s exact test was used when the conditions for using the chi-square test were not met. A 5% significance level was used to evaluate the statistical tests.

Data were input to an Excel spreadsheet and the program used to perform statistical calculations was IMB SPSS version 25.

## RESULTS

103 fourth-year medical students participated in the study, 69 women and 34 men. The vast majority had no experience with USG-guided puncture (99 students). Only 4 students had previously participated in such procedures, 2 during central venous access, 1 in a thyroid nodule puncture, and 1 in a liver biopsy.

**Figure 2:**
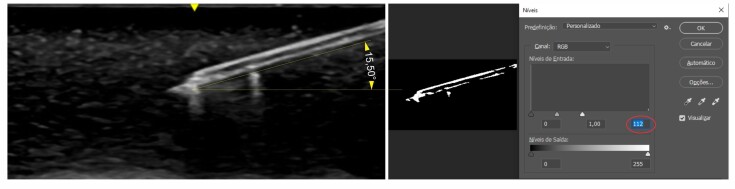
Image containing 112 pixels, showing the needle at 15.51º in relation to the surface - Considered satisfactory by the students.

**Figure 3:**
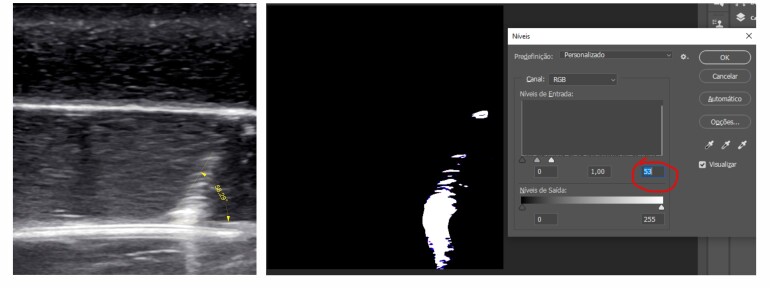
Image containing 53 pixels, showing the needle at 58.29º in relation to the surface with- Considered unsatisfactory by the students.

The approximate percentage distribution of images classified as sufficient and insufficient was 48.5% sufficient and 51.5% insufficient. The comparison between the 20 images observed by the 103 students and those evaluated using photoshop showed that images with more than 60 pixels had a higher percentage of sufficient ratings (72.1% vs. 4.6%). Images acquired with a puncture angle less than 45º also had a higher percentage considered sufficient by the students (69.8% vs. 16.5%) and there were significant associations between the students’ classifications and the number of pixels and image angle (p < 0.001) (Table 1) (Figures 2 and 3).

**Table 1:** Classifications of 2060 evaluations of 20 images by 103 students according to number of pixels and puncture angle.

Variable	Students’ classifications		TOTAL n (%)	p-value
	Insufficient	Sufficient		
	n (%)	n (%)		
Whole group	**1061 (51.5)**	**999 (48.5)**	**2060 (100.0)**	
Number of pixels				**p**(1) < 0.001*
< 60	688 (95.4)	33 (4.6)	721 (100.0)	
> 60	373 (27.9)	966 (72.1)	1339 (100.0)	
Angle				p^(1)^ < 0.001*
< 45	373 (30.2)	863 (69.8)	1236 (100.0)	
> 45	688 (83.5)	136 (16.5)	824 (100.0)	

* Association significant to 5%.

^(1)^Pearson’s chi-square test.

When evaluating 10 images taken using a IV catheter and 10 images taken using the introducer needle, a higher percentage of images in the IV catheter group were considered sufficient by the 103 students (54.3% vs. 42.7%). Regarding the needle visualization software, students considered a higher percentage of images using the software sufficient than images that did not use the software (57.9% x 39.1%), with a significant association between the results of the 103 students’ classifications and each of the groups analyzed (p < 0.001) (Table 2).

**Table 2:** Classifications of 2060 evaluations of 20 images by 103 students according to the needle used for puncture and use of software to view the needle.

Subgroup	Students’ classifications		TOTAL n (%)	p-value
	Insufficient	Sufficient		
	n (%)	n (%)		
Whole group	**1061 (51.5)**	**999 (48.5)**	**2060 (100.0)**	
IV catheter	471 (45.7)	559 (54.3)	1030 (100.0)	**p**(1) < 0.001*
Introducer needle	590 (57.3)	440 (42.7)	1030 (100.0)	
Without software	627 (60.9)	403 (39.1)	1030 (100.0)	p^(1)^ < 0.001*
With software	434 (42.1)	596 (57.9)	1030 (100.0)	

* Association significant to 5%.

^(1)^Pearson’s chi-square test.

## DISCUSSION

Adequate visualization of the needle on USG is extremely important for successful puncture, whether of a solid structure (thyroid, liver, kidney) or for vascular access or anesthetic blockade.^2^

Our study identified that even people with little or no experience were able to identify satisfactory images for performing a puncture when compared with the quality of images according to Photoshop. Studies that compared the relationship between the perception of the human eye and what was observed using computer programs found similar results.^8,9^

The angle of needle insertion in relation to the surface and consequently in relation to the beam of sound waves emitted by the ultrasound probe is very important for obtaining an adequate image. Punctures of superficial structures tend to have better visualization, since they penetrate the surface at an angle that is closer to perpendicular to the wave beam, reflecting more of the sound waves for image formation. At the same time, punctures of deeper structures that require a steeper puncture in relation to the surface tend to have a lower quality image, because there will be greater dispersion of sound waves, impairing image formation.^7,14,15^ The students observed this effect, classifying as satisfactory images those showing punctures at angles smaller than 45º in relation to the surface and as unsatisfactory when punctures were at angles greater than 45º.

The number of pixels reflects the quality of the image. The more pixels, the better the image quality. This confirmed what the students classified as being sufficient or insufficient. In other words, the more pixels were observed in an image on photoshop, the more students classified it as being sufficient. This was expected in advance, but we thought it important to avoid any confounding bias introduced by the students.

The angle effect can be compensated for by using needle visualization software. With this feature, the equipment can redirect the sound beam towards the needle, improving sound reflection back to the probe and providing a better image.^9,16^ This is especially important in situations when a steeper puncture is necessary in relation to the surface, i.e., in situations in which the structure to be punctured is at a deeper position.

The material from which the needle is manufactured also affects USG image formation. Studies have observed that materials with specific coatings and surface designs can generate better images by reflecting more sound waves.^8,17,18^

The human ability to align the needle and the probe at the time of puncture is perhaps still the main factor in formation of adequate USG images.^2^ However, it is possible to reduce the human factor in these cases, observing the way the images are formed on the USG.

The main limitations of our study include use of a gelatin model to perform the punctures, which facilitates visualization of the needle, in view of the large difference in impedance between the needle and its surroundings. Additionally, few needle models were used for comparison purposes, no well-defined subjective criteria were applied to identify images as satisfactory or unsatisfactory by the human eye, and comparison with a control group of specialists was lacking.

## CONCLUSIONS

Even in people with little or no experience, the human eye is capable of identifying satisfactory images for USG-guided puncture. In addition, visualization of the needle is best obtained at angles smaller than 45º in relation to the surface, using a siliconized stainless steel needle, and with specific software for needle recognition.
